# Single‐cell RNA‐sequencing of retrieved human oocytes and eggs in clinical practice and for human ovarian cell atlasing

**DOI:** 10.1002/mrd.23648

**Published:** 2022-10-20

**Authors:** Jordan H. Machlin, Ariella Shikanov

**Affiliations:** ^1^ Program in Cellular and Molecular Biology University of Michigan Ann Arbor Michigan USA; ^2^ Department of Biomedical Engineering University of Michigan Ann Arbor Michigan USA; ^3^ Department of Obstetrics and Gynecology University of Michigan Ann Arbor Michigan USA

**Keywords:** human ovary, single‐cell RNA‐sequencing, transcriptomics

## Abstract

With the advancement of single‐cell separation techniques and high‐throughput sequencing platforms, single‐cell RNA‐sequencing (scRNA‐seq) has emerged as a vital technology for understanding tissue and organ systems at cellular resolution. Through transcriptional analysis, it is possible to characterize unique or rare cell types, interpret their interactions, and reveal novel functional states or shifts in developmental stages. As such, this technology is uniquely suited for studying the cells within the human ovary. The ovary is a cellularly heterogeneous organ that houses follicles, the reproductive and endocrine unit that consists of an oocyte surrounded by hormone‐producing support cells, as well as many other cell populations constituting stroma, vasculature, lymphatic, and immune components. Here we review studies that have utilized scRNA‐seq technology to analyze cells from healthy human ovaries and discuss the single‐cell isolation techniques used. We identified two overarching applications for scRNA‐seq in the human ovary. The first applies this technology to investigate transcriptional differences in oocytes/eggs from patients undergoing in vitro fertilization treatments to ultimately improve clinical outcomes. The second utilizes scRNA‐seq for the pursuit of creating a comprehensive single‐cell atlas of the human ovary. The knowledge gained from these studies underscores the importance of scRNA‐seq technologies in unlocking a new biological understanding of the human ovary.

## 1 INTRODUCTION

Characterizing the single cells in an organ and understanding their individual functions provides the foundational significance of RNA sequencing technologies. Next‐generation RNA‐sequencing has been widely used to profile the transcriptome of bulk tissue. Now, due to the advancement of single‐cell collection methodologies, it is possible to determine gene expression at the single‐cell level by measuring an individual cell's transcriptome. This process, known as single‐cell RNA‐sequencing (scRNA‐seq), combines high‐throughput sequencing platforms with bioinformatics analysis to provide a readout of cellular transcriptomes. The single‐cell transcriptome is a molecular signature that encompasses the genes transcribed by each cell and their level of transcriptional output. Cellular identity is more broadly defined by a variety of factors influencing the cell at any given time such as its environmental milieu, cell cycle stage, position in a developmental lineage, and external stimuli (Wagner et al., 2016). Revealing the transcriptional networks in an organ system, at the single cell level, can be important both biologically and translationally because scRNA‐seq has the potential to distinguish the unique cells present, improve our understanding of cellular differentiation trajectories and identify the innate developmental patterns of cells. Emergence of this technology has provided great insights into the cellular makeup of several organ systems throughout the human body including the lung, kidney, liver, and thymus (Liao et al., 2020; MacParland et al., 2018; Park et al., 2020; Travaglini et al., 2020).

Recently, studies have utilized scRNA‐seq technology on individual cells from the human ovary to further understand this complex organ. The ovary is the source of fertility and endocrine function in females. It contains follicles comprised of an oocyte surrounded by steroidogenic somatic cells, stromal cells, blood vessels, immune cells, and extracellular matrices. Cohorts of dormant primordial follicles continuously activate and enter the growing pool where they begin to produce hormones that have systemic effects throughout the entire body (Fuentes & Silveyra, 2019). This organ is cellularly heterogeneous, making scRNA‐seq an excellent approach for studying its complexity. Here we reviewed studies that have utilized scRNA‐seq on human ovarian cells. We performed a PubMed‐based literature review using the key words “Single Cell RNA Sequencing Ovary” and “Single Cell Transcriptomics Ovary and Oocytes” and selected the species “humans” which resulted in a total of 210 reports. After screening the titles of these papers, 40 were deemed relevant. Of those, six fit into our inclusion criteria. Our inclusion criteria allowed any paper performing scRNA‐seq on any cell type in healthy non‐diseased human ovaries. We excluded studies utilizing tissue from patients with a history of cancer, differences in sex development (DSDs), endometriosis, polycystic ovary syndrome (PCOS), or obesity. We also included nine articles previously identified that did not come up in the search terms. In total, we reviewed 15 papers that fit into our inclusion criteria to discuss the use of scRNA‐seq technology in the human ovary. Comparisons of single‐cell sequencing platforms and optimal bioinformatics analysis methods for this technology have been reviewed extensively elsewhere (AlJanahi et al., 2018; Kanter & Kalisky, 2015; Kolodziejczyk et al., 2015; Wagner et al., 2016; Ziegenhain et al., 2017).

## 2 SINGLE‐CELL TRANSCRIPTOMICS IN THE HUMAN OVARY

### 2.1 Single‐cell RNA‐sequencing workflow and applications in human ovarian research

The typical workflow of scRNA‐seq experiments starts with the high‐yield separation of single cells from a bulk population (AlJanahi et al., 2018). Many advancements have been made for dissociating individual cells while keeping RNA quality and cellular structural integrity intact (Nguyen et al., 2018). These include: manual isolation (Tang et al., 2009), microfluidic droplet‐based devices (Lareau et al., 2019; Thorsen et al., 2001), capillary electrophoresis (Butler, 2012), laser capture microdissection (Datta et al., 2015), magnetic activated cell sorting (MACS; Bacon et al., 2020) and fluorescence‐activated cell sorting (FACS; An & Chen, 2018; Basu et al., 2010). Many of these separation methods can be used for isolating cells in the human ovary for sequencing constituting a range of manual difficulty, time consumption, and tissue fixation requirements (Table 1). Determining the technique to use is largely based on the cell population of interest. For example, targeted separation techniques such as FACS and MACS are used to enrich a population of cells that express cell surface markers of interest (like a germ cell marker or hormone receptor) or to ensure the population of cells to be sequenced are live (Fan et al., 2019; Li et al., 2017; Man et al., 2020; Wagner et al., 2020). Laser‐capture microdissection (LCM) is a technique used in RNA sequencing protocols whereby cells of interest are cut from a fixed tissue section using a UV laser followed by RNA extraction (Datta et al., 2015). Typically, LCM has been used to isolate whole tumors and their associated cells compared to control regions of a fixed histology slide for transcriptomic characterization (Gómez‐Cuadrado et al., 2022; Ong et al., 2020). This technique has been co‐opted for use in the ovary to collect single oocytes for RNA sequencing (Ernst et al., 2017). This is possible because of the distinct morphological structure of ovarian follicles and the large size of human oocytes which allows a relatively precise isolation with the UV laser (Ernst et al., 2017). LCM is useful for isolating cells within the context of their native environment, however, it is based on morphological appearance which can be subjective and requires tissue fixation, which could alter RNA integrity (Srinivasan et al., 2002).

**Table 1:** Single‐cell isolation techniques used in the human ovary

Technique	Description	Infographic	Targeted or untargeted	Fixed or fresh	Limitations	scRNA‐seq paper using the technique
Direct cell lysis (DCL)	Researcher mechanically isolates single cells of interest and places them directly into a lysis or storage buffer.	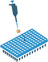	Untargeted	Fresh	Technically the most challenging and time‐consuming Not ideal for “whole tissue” atlasing	Li et al. (2017) Y. Zhang et al. (2018) Fan et al. (2019) Fan et al. (2021, 2021) a
Fluorescence‐activated cell sorting (FACS)	Specialized flow cytometry. Sorts heterogeneous cell mixture one cell at a time based on light scattering and fluorescent characteristics of the cell. Used in conjunction with DCL	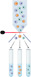	Targeted	Fresh	Requires a cell surface marker for separation so excludes rare cell types Bulk groups of cells are isolated so an additional method of individual isolation is needed	Li et al. (2017) Fan et al. (2019) Man et al. (2020) Wagner et al. (2020)
Magnetic‐activated cell sorting (MACS)	Target cells are tagged with magnetic particles bound to antibodies and passed through a magnetic field. Isolates cells into bulk groups. Using in conjunction with DCL	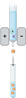	Targeted	Fresh	Requires a cell surface marker for separation so excludes rare cell types Bulk groups of cells are isolated so an additional method of individual isolation is needed	Li et al. (2017)
Laser‐capture microdissection (LCM)	Tissue is paraffin‐embedded and sectioned. Outline of cells of interest is marked and cut using a UV laser. Cells are lifted onto a sterile cap for RNA collection.	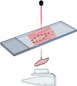	Untargeted	Fixed	Tissue fixation could alter/damage RNA quality Imprecise and subjective method of cutting out cells of interest	Ernst et al. (2017)

^a^ All improvement of IVF outcomes papers used the DCL method. Infographics created in BioRender.com.

The limitations of LCM can be avoided by isolating cells from live tissue which is accomplished through a technique called direct cell lysis (DCL). In this method, ovarian tissue is enzymatically digested and individual cells of interest are manually collected by micropipette and placed directly into lysis buffer in preparation for sequencing. The DCL method is used in the human ovary because it overcomes a few major challenges of scRNA‐seq involving the isolation of individual oocytes due to their size and rarity. One challenge has to do with the channel diameter of droplet‐based microfluidic devices typically used to separate a bulk suspension into individual cells before sequencing. The leading manufacturing company of single‐cell sequencing devices, 10× genomics, reports that cells larger than 40 µm can clog their devices (10x Genomics, n.d.). This is a problem because depending on their developmental stage, human oocytes that reach diameters greater than 40 µm may get stuck in the channels and are unable to be sequenced (Lintern Moore et al., 1974). DCL also addresses the challenge that arises when researchers want to sequence the individual components of the follicle, the oocyte, and somatic cells, separately. In general, to obtain a suspension of cells for sequencing, tissue pieces are enzymatically digested to dissociate individual cells before being processed through a sequencing device. From our experience, it is difficult to isolate the oocyte from its surrounding cells using enzymatic digestion alone as they maintain a close association, especially in earlier stages. Instead, following digestion, follicles must be mechanically manipulated to release their oocytes. From there, oocytes need to be individually placed into wells containing lysis buffer for sequencing. For these reasons, DCL is the only feasible isolation method that can be used to obtain individual oocytes for RNA‐sequencing. Therefore, implementing this process is integral for understanding the cellular transcriptome of this organ.

Despite some of the technical limitations of using scRNA‐seq in the human ovary, application of scRNA‐seq has been critical to many clinical and basic science areas of study and its use can largely be categorized into two different goals. The first clinically translational goal applies this technology to investigate the transcriptome of single cells obtained during ovarian stimulation cycles to identify transcriptional differences between retrieved oocytes/eggs and investigate factors that might impair or disrupt normal fertilization and IVF cycle success. The second is driven by basic biological inquiries aiming to create a single‐cell atlas of human ovarian cell types and understand what controls follicle atresia and activation. These goals for using scRNA‐seq in the ovary have provided an in‐depth understanding of the cell types involved and molecular mechanisms required for proper ovarian function.

### 2.2 scRNA‐Seq for the improvement of IVF outcomes

**Figure 1:**
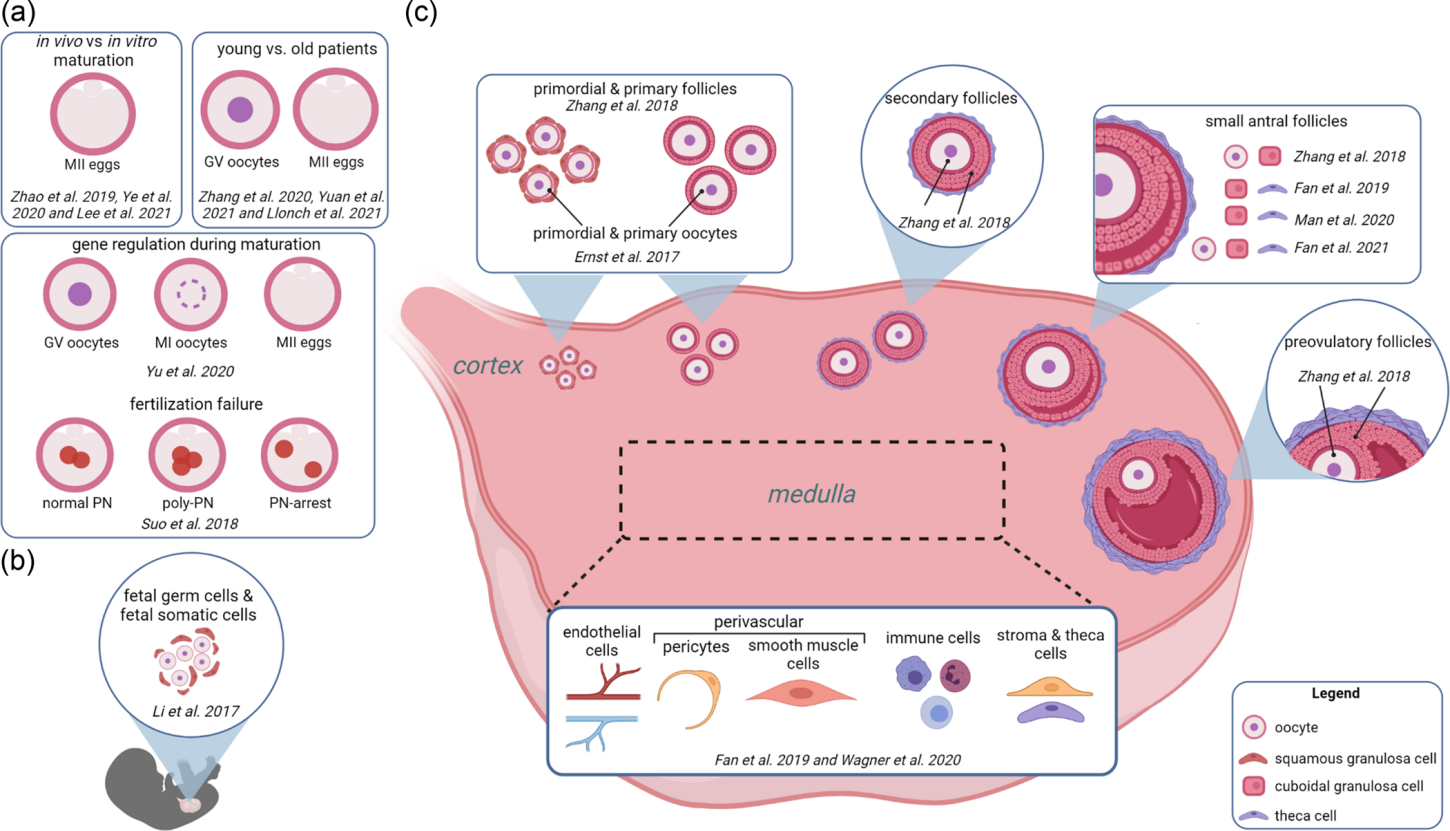
scRNA‐seq experiments analyzed oocytes, eggs, and ovarian cells collected from various sources. (a) scRNA‐seq analysis on oocytes, eggs, and fertilized eggs for clinical purposes. (b) scRNA‐seq analysis performed on fetal germ cells and their niche cells from 4 to 26 weeks post‐fertilization. (c) scRNA‐seq analysis on cells from the cortex and medulla in reproductive age women. Created in BioRender.com.

IVF cycle success is primarily defined by the outcome of a live birth (Min et al., 2004; Rienzi et al., 2021). Cycle success depends on a multitude of factors including but not limited to: maternal age, patient history, number of fertilizable eggs, and total number and quality of embryos (Amini et al., 2021; Totonchi et al., 2021). scRNA‐seq is a useful tool to measure transcriptomic differences that may arise in oocytes, eggs, and embryos that are affected by these diverse factors to ultimately provide avenues that could improve IVF cycle success. Generally, scRNA‐seq technology for the improvement of IVF outcomes has been applied for three broad goals: to determine changes in gene expression during artificial (in vitro) oocyte maturation compared to in vivo matured eggs, to correlate maternal age‐related differential gene expression and egg quality, and to decipher the molecular mechanisms of fertilization and oocyte maturation failure (Figure 1a).

### 2.3 Changes in gene expression during in vitro maturation

IVF procedures simulate the hormonal trigger needed for oocyte meiotic maturation to the MII stage. This is accomplished through the delivery of an LH‐homologous hormone, called human chorionic gonadotropin (hCG), that facilitates meiotic resumption and retrieval of matured MII eggs in clinic (Abbara et al., 2018). However, despite the stimulation with hCG, up to 30% of oocytes collected during IVF cycles can still be in an immature—either germinal vesical (GV) or metaphase I (MI)—stage (Cha & Chian, 1998; De Vos et al., 1999; Huddleston et al., 2009; Jee et al., 2008; H. J. Lee et al., 2012; Mandelbaum et al., 1996; Moore et al., 2007; Nogueira et al., 2006). In humans, eggs are fertilizable only at the MII stage of meiosis after they have extruded the first polar body (Voronina & Wessel, 2003). Therefore, clinics have been attempting a rescue in vitro maturation (rIVM) to mature oocytes retrieved in an immature state, increasing the number of potentially fertilizable eggs available for patients. Overall, there is a limited understanding of whether the process of IVM leads to changes in gene expression that would not occur during typical in vivo maturation (IVO). Furthermore, the IVM media formula originates from medical in vitro maturation (mIVM) protocols which are performed on the entire cumulus–oocyte complex (COC) from ovaries that have not undergone ovarian stimulation in cases where ovarian hyperstimulation syndrome (OHSS) is a concern (Chian et al., 2013; A. W. T. Lee et al., 2021). This media contains substrates to support the high metabolic requirements of the cumulus cells which in the case of rIVF in a clinical setting would be fully removed to assess maturation status (Gilchrist et al., 2011). For these reasons, researchers are utilizing scRNA‐seq to determine the key genes in oocytes/eggs that are altered during the process of both rIVM and mIVM (A. W. T. Lee et al., 2021; Ye et al., 2020; Zhao et al., 2019).

In 2019, Zhao et al. (2019) used single‐cell technology to characterize the transcriptome of human eggs matured in vitro and in vivo. The study compared mature (MII) eggs and immature (GV) oocytes after retrieval from three donors. The immature oocytes underwent rIVM and were selected for sequencing after expelling a polar body. Sequencing was performed on six eggs total, three in vitro matured (rIVM) and three in vivo matured (IVO). After principal component analysis (PCA) the rIVM and IVO eggs clustered separately, revealing 2230 differentially expressed genes (DEGs). Out of these DEGs, the most highly affected pathways in rIVM eggs were related to metabolism and cell signaling. Expression of the mitochondrial genes *ACAT1* and *HADHA* was significantly downregulated in rIVM eggs. The deficiency of these two metabolic pathway genes in rIVM eggs compared to IVO eggs and the lower expression of genes involved in the Krebs cycle led researchers to conclude that there is disrupted energy metabolism in the rIVM eggs. Furthermore, their data indicated the expression of *DPYD* to be significantly upregulated in rIVM eggs. This gene plays a key role in repair of double‐strand breaks (DSBs) in DNA. With its higher expression in the rIVM eggs, this suggested a possible compensatory mechanism for an increase in DSBs in the in vitro environment. They conclude that energy metabolism and proper mitochondrial function are the limiting factors in proper rIVM egg developmental potential and targeting these processes could be an option to improve outcomes of rIVM egg use in clinic.

A second study by Min Ye et al. (2020), utilized scRNA‐seq to investigate transcriptomic differences in eggs after medical in vitro maturation (mIVM) compared to in vivo matured eggs (IVO). They collected COCs from large antral follicles and subjected them to mIVM. Overall, seven mIVM and eight IVO matured MII eggs were used for scRNA‐seq analysis. The DEGs with higher expression in IVO matured eggs were enriched for pathways involving cell cycle, mRNA metabolism, and DNA metabolism. The DEGs with higher expression in mIVM matured eggs included pathways participating in mitochondrial respiratory chain biogenesis, ER stress, and ATP metabolism. This group hypothesized that differential DNA methylation might contribute to the DEGs in the mIVM eggs. However, the whole genome CpG methylation was indistinguishable between the two groups. From there, they explored the differentially methylated regions (DMRs) of the genome which are regions that are typically associated with transcriptional regulation. Here they found differences between the two groups in the DMRs which could potentially affect the transcriptional regulation of specific genes. Finally, they examined the copy number variant (CNV) of IVO and mIVM eggs to determine their ploidy status and determined that aneuploidy did not affect the transcript level. Interestingly, despite the differential expression between the two groups several mIVM eggs have been fertilized and developed into healthy embryos and live births suggesting that mIVM eggs could potentially tolerate the DEGs. Thus, they conclude the DEGs minorly impact mIVM eggs and there is a need for more long‐term studies to determine if there are negative consequences on embryo development and live birth rate when mIVM eggs are used.

Most recently, A. W. T. Lee et al. (2021) performed scRNA‐seq on mature eggs collected after rescue IVM (rIVM) and in vivo (IVO) matured eggs, analyzing 10 eggs from each group. The rIVM and IVO eggs formed distinct clusters and 1165 upregulated and 394 downregulated DEGs were identified in rIVM eggs. Network pathways analysis revealed that the top process to be most significantly different between rIVM and IVO eggs was mitochondrial translation with DEGs significantly upregulated in rIVM eggs. They explored the transcriptional regulatory pathways that might drive the DEGs in rIVM eggs. The most significant regulatory pathways were ETS Proto‐Oncogene I (*ETS‐I*) and GATA‐binding factor I (*GATA‐I*) where expression of signaling genes in these pathways was reduced in rIVM eggs. The genes controlled by these pathways are involved in energy metabolism and antiapoptosis. Therefore, reduced regulation of these two signaling pathways in rIVM eggs might affect egg metabolism and stress responses. The authors of this paper compared their data set with that of Zhao et al. and Ye et al. (discussed above). Contrary to Zhao's group, they found that expression of *ACAT1* and *HADHA* was elevated in the rIVM eggs compared to IVO eggs. Their data overlaps with Ye et al.'s work which showed that mIVM eggs demonstrated an upregulation of mitochondrial translation activities which was also seen in the rIVM eggs of this study. Overall, A. W. T Lee's research group revealed specific genes that could reduce the performance of rIVM eggs. They conclude by arguing the need for IVM media that is optimized specifically for rescue IVM in oocytes without surrounding cumulus cells before incorporating this technique in an IVF practice.

In summary, although the results from these three reports differed with respect to which genes were upregulated or downregulated, overall, there was a consensus that energy metabolism was dysregulated in eggs that underwent in vitro maturation.

### 2.4 Maternal age and egg quality

Advancing maternal age, which is clinically defined as 35 years old or older, contributes to a decline in oocyte and egg quantity and quality (Liu et al., 2011). This phenomenon has been widely studied and reviewed with considerations such as chromosomal factors, diminishing ovarian reserve, increasing oxidative stress, metabolic dysregulation, and microenvironmental changes (Crawford & Steiner, 2015; Mikwar et al., 2020; Park et al., 2021; Wang et al., 2021). Using scRNA‐seq to compare the transcriptome of oocytes and eggs retrieved from young vs older patients undergoing IVF treatments may help understand the molecular mechanisms underlying the deterioration of oocyte and egg quality with age (Llonch et al., 2021; Yuan et al., 2021; J.‐J. Zhang et al., 2020).

The first of these studies utilizing scRNA‐seq to look for maternal age effects was performed by J.‐J. Zhang et al. (2020) whose group compared three younger (27.0 ± 1.0 years) and three older (43.3 ± 2.1 years) patients that donated one MII egg for sequencing. They identified 149 downregulated genes and 208 upregulated genes between young and old patient eggs. Specifically, they interrogated a set of seven genes that showed a significant downregulation in older eggs. Among them, *TOP2B*, a chromatin structure and gene expression regulation‐related genes were chosen to be investigated further. By knocking down *TOP2B* expression in mature eggs from young mice and fertilizing them, embryos arrested at the two‐cell stage of development provide evidence that this gene is essential for early embryogenesis. Additionally, the researchers found two genes associated with DNA damage repair *RAD50* and *RAD17* to be downregulated in eggs with age. This finding indicates that eggs might have an impaired ability to combat DNA damage as they age. They also point to an interesting finding that the most enriched gene ontology (GO) term among the genes downregulated with maternal age was catalytic activity whereas in genes that were upregulated with age there was an enrichment in transcriptional activation.

Following this study, Yuan et al. (2021) interrogated egg transcriptomic changes with age using single‐cell analysis in 12 MII eggs. Six eggs were donated by younger women (26.83 ± 1.94 years) and six were donated by older women (42.67 ± 2.25 years). It is important to note that only three MII eggs from the younger population were included in the analysis. The reason for this was twofold, these three younger eggs did not cluster separately from the older egg population. Upon further investigation into the clinical data, the three eggs that were excluded came from patients that showed significant differences in basal FSH, MII transition rate, and percentage of available embryos when compared to the other three patients whose eggs were included. Overall, they identified 322 upregulated and 159 downregulated genes with advancing age. Compared to the previous study their gene enrichment analysis of the differentially expressed genes pointed to changes in transcription, ubiquitination, cell cycle oxidative phosphorylation, and oocyte meiosis. Specifically, genes involved in oxidative phosphorylation and ATP synthesis—important processes for oocyte maturation—were downregulated in older eggs. This finding is consistent with the emerging hypothesis that mitochondrial dysfunction plays a role in ovarian aging in part due to oxidative phosphorylation dysregulation (Amorim et al., 2022; Kasapoğlu & Seli, 2020). Additionally, the DEG enrichment analysis indicated significant differences in the regulation of ubiquitination between young and old eggs with a mix of up‐ and downregulated genes suggesting the balance of ubiquitination is disrupted with age. This is important because protein degradation by the ubiquitin‐proteosome system (UPS) plays a role in oocyte maturation and mammalian fertilization and embryogenesis (Sutovsky, 2003). Furthermore, genes involved in spindle formation and chromosome segregation were influenced by egg age which reinforces the higher incidence of aneuploidy found in older eggs.

The final study investigating aging was by Llonch et al. (2021). Their study used a patient population of 37 women between 18 and 43 years old with the young group defined as <35 and the old group defined as >35. The difference here is that they collected 72 oocytes and eggs, 40 oocytes in the GV stage and 32 MII eggs post IVM (IVM‐MII). They were interested to see if age is a differentiating feature within each maturation stage but found that the maturation stage was the main differentiator of oocyte and egg transcriptomes. Instead, they found distinct sets of genes that showed altered RNA expression during aging within the GV population and IVM‐MII populations separately. Among the few genes that showed alterations in age in both maturation stages, the gene *ND1* was downregulated with age. This gene is involved in electron transport chain in mitochondria and agrees with the overall decrease in oxidative phosphorylation and ATP synthesis seen in the previous data set by Yuan et al. Another finding consistent with the study from Yuan et al. is aging had an impact on the transcript representation of genes related to chromosome segregation. Finally, researchers found genes related to oxidative stress protection to decrease with age in both populations.

These three data sets provide important transcriptomic factors that are altered in oocytes and eggs with advancing age. All three indicate that changes in transcriptional activity may be dysregulated in older eggs. Additionally, eggs from older patients showed impairments in oxidative stress, DNA damage repair, and chromosome segregation which suggests an impaired ability to combat DNA damage.

### 2.5 Fertilization failure and gene regulation across stages of oocyte maturation

Fertilization fails in 5%–10% of IVF cycles and in 2%–3% of intracytoplasmic sperm injection cycles (Mahutte & Arici, 2003). Unfortunately, couples who experience failed fertilization during an IVF cycle are ~30% more likely to have failed fertilization recur in subsequent cycles (Kahyaoglu et al., 2014). Furthermore, the proper maturation of human oocytes across GV, MI, and MII stages is imperative for successful fertilization.

To understand the gene regulatory dynamics involved in oocyte maturation Yu et al. (2020) generated single‐cell transcriptome data from oocytes and eggs at three maturation stages; GV, MI, and MII. These oocytes and eggs were collected from 17 women of reproductive age undergoing retrieval for assisted reproductive technologies. Interestingly, after analyzing the top 1000 expressed genes among these three stages, the GV and MI oocytes clustered separately from eggs at the MII stage. There were no DEGs between MI and GV oocytes suggesting they have similar gene expression at these stages. However, many DEGs were observed between MII eggs and the immature stages. This group focused on the MII/MI DEGs because they were interested in the transition from immature oocytes to mature eggs. The highly upregulated genes in MII eggs were involved in RNA degradation, splicing, and transport whereas the downregulated genes fell into pathways involving the Krebs cycle and oxidative phosphorylation. They explain that the downregulation of RNA‐related pathways makes sense in MII eggs because there is slow maternal RNA degradation during oocyte maturation but once fertilized, maternal RNA is rapidly degraded so mature eggs could be preparing for this event. Furthermore, the downregulation of Krebs cycle and oxidative phosphorylation pathway genes has been confirmed by previous studies that have shown alternative glucose metabolism pathways in the oocyte cytoplasm during maturation (Collado‐Fernandez et al., 2012; Dumollard et al., 2006; Sutton et al., 2003; Sutton‐McDowall et al., 2004).

To clarify the molecular mechanisms of fertilization failure at the single‐cell level, L. Suo et al. (2018) profiled the transcriptomes of abnormally fertilized zygotes from two patients with recurrent total fertilization failure (RTFF). Following ovarian stimulation and retrieval, eggs were fertilized and resulting zygotes were graded for abnormalities. Normal fertilization was indicated by the presence of two pronuclei (one from egg and sperm) 16–18 h post insemination. Zygotes with more than three pronuclei (poly‐PN zygotes) and zygotes with normal pronuclei that failed to fuse hours after fertilization (PN‐arrest) were used for scRNA‐seq. The poly‐PN zygotes had defects in meiosis and RNA processing whereas the PN‐arrest zygotes had defects in cell cycle and DNA homologous recombination. Zygotes with multiple pronuclei after fertilization have difficulty undergoing complete meiosis. Genes associated with segregating chromatids and progression of meiosis were dysregulated in the poly‐PN zygotes. PN‐arrested zygotes showed downregulation of several genes related to the cell cycle machinery involved in the shift from meiotic to mitotic chromosome segregation, which is critical for normal embryo development (Clift & Schuh, 2013). Through scRNA‐seq this paper identifies several gene pathways that are disrupted in abnormally fertilized zygotes that have the potential to be targeted for therapeutic intervention in the future.

In summary, these findings together suggest the importance of increased transcription of meiosis and cell cycle‐related genes during oocyte maturation to prepare for normal fertilization.

### 2.6 scRNA‐Seq for the creation of a single cell atlas

In addition to its clinical application, single‐cell sequencing technology is used to create a complete cellular map of ovarian cell types (Ernst et al., 2017; Fan et al., 2019, 2021; Li et al., 2017; Man et al., 2020; Wagner et al., 2020; Y. Zhang et al., 2018). Cell clustering based on known marker genes reveals the unique transcriptomes and molecular mechanistic differences between various ovarian cells that are involved in folliculogenesis, steroidogenesis, ovulation, remodeling, and immune functions (Ernst et al., 2017; Fan et al., 2019, 2021; Li et al., 2017; Man et al., 2020; Wagner et al., 2020; Y. Zhang et al., 2018). We have reviewed seven single‐cell sequencing papers that have taken significant strides to characterize the transcriptomes of cell types in the human ovary (Ernst et al., 2017; Fan et al., 2019, 2021; Li et al., 2017; Man et al., 2020; Wagner et al., 2020; Y. Zhang et al., 2018; Figure 1b,c).

Li et al. (2017) performed a comprehensive analysis of female fetal germ cells (FGCs) and their niche cells, assessing their cellular identity and identifying crucial transcription factors of stage‐specific development. Their transcriptomic analysis revealed four subpopulations of female FGCs: mitotic phase, RA signaling‐responsive phase, meiotic prophase, and oogenesis phase. Researchers identified receptor–ligand pairs for the BMP and Notch signaling pathways in the female gonadal somatic cells and FGCs. Importantly, this study group identified candidate transcription factor regulators that may play a crucial role in the coordination of stage‐specific development.

To describe the gene activity and canonical pathways of oocytes in the primordial to primary stage transition, Ernst et al. (2017) utilized scRNA‐seq on pools of LCM‐isolated primordial and primary oocytes from three patients. They found that *FOXO1*, the downstream transcription factor of the PI3K/AKT pathway, was significantly upregulated in primordial oocytes, whereas *EIF4E*, a critical oocyte development gene was upregulated in primary oocytes (Ernst et al., 2017). This study provides an in‐depth pathways analysis describing genes that are differentially up‐ or downregulated during the transition from quiescent primordial to activated primary oocytes. These genes can be investigated further to elucidate some of the mechanisms behind primordial follicle activation.

Fan et al. (2019) utilized scRNA‐seq on manually selected tissue samples containing stroma and visible antral and atretic follicles from the ovarian medulla to identify the somatic cell types in the adult ovary. Cells are clustered in five major types including granulosa, theca/stroma, smooth muscle, endothelial and immune cells. Further analysis of gene expression revealed an increase in the complement system gene *C1S* in healthy compared to degenerating follicles and an overabundance of CD68+ macrophages in degenerating follicles suggesting an innate immune response potentially involved in follicular remodeling. Other complement system genes they characterized included *C1R, C7*, and *SERPING1* which reflect immune cells resident to the adult ovary.

To understand the ovarian single‐cell landscape more completely, Wagner et al. (2020) combined the Fan group's data set on the medulla with their single‐cell data set from the ovarian cortex. Using cortical tissue from patients undergoing gender reassignment surgery who had been on long‐term androgen therapy, Wagner et al. identified five clusters: immune, granulosa, endothelial, perivascular, and stroma which overlapped with Fan's data set. Their group identified a cluster of oocytes in the data set that was not seen in Fan's that likely came from small primordial follicles residing in the ovarian cortex. Although the focus of this paper was to disprove the presence of adult ovarian stem cells, they also provided a more well‐rounded view of the molecular signatures in the ovarian landscape.

Several other sequencing papers have tried to elucidate the transcriptomic signatures of oocytes and their surrounding somatic cells across a range of follicular developmental stages (Fan et al., 2021; Man et al., 2020; Y. Zhang et al., 2018). The first of these by Y. Zhang et al. (2018) profiled five stages of follicles between 50 and 300 µm in diameter and provided gene signatures for each follicle stage. They performed an interesting analysis of the gap junctions and signaling pathways involved in the cross‐talk between oocytes and granulosa cells using matched GC and oocyte samples and found specific connexin‐encoding genes that localized in distinct follicle stages (Y. Zhang et al., 2018). Another study by Man et al. (2020) performed scRNA‐seq on antral follicles between 1 and 3 mm that were of ovarian or xenograft origin. This study did not include any oocytes in their analysis but instead concentrated on the granulosa and theca cell populations (Man et al., 2020). The analysis revealed several cell surface markers, such as *CD99*, *CD55*, *ENG*, and *ANPEP*, that distinguish granulosa cells from theca cells (Man et al., 2020). Additionally, they determined the surface marker *PVRL1* distinguishes cumulus versus mural granulosa cells—the two granulosa cell compartments within the antral follicle (Man et al., 2020). This study provides important cell surface markers that can be used to isolate or target discrete compartments of the follicle for future studies. To round out the transcriptomic profile of larger follicles, Fan et al., performed scRNA‐seq on oocytes and their surrounding cumulus granulosa cells in antral follicles 1–8 mm in diameter (Fan et al., 2021). They compared this data set with their previous analysis (Fan et al., 2019) and showed that, interestingly, the cumulus granulosa cells in this data set did not separate from the mural granulosa cells in the antral follicles. This indicates that these two granulosa cell compartments show similar molecular signatures.

These papers have contributed greatly to our understanding of the cell types present in the human ovary and the molecular signatures of unique compartments and follicle stages. However, scRNA‐seq in the human ovary has some limitations that are demonstrated in these papers. While extremely beneficial, human tissue studies are limited by donor tissue availability and small samples sizes. Some of the patients whose tissue was analyzed were cancer patients undergoing elective ovariectomy (Fan et al., 2019) or were from patients that had been on long‐term androgen therapy (Wagner et al., 2020). These diagnoses and treatments could have unknown consequences on the ovary and have the potential to introduce transcriptome differences. For example, our work with a male transgender mouse model identified significant upregulation of genes associated with inflammation (Kinnear et al., 2019, 2021). Furthermore, though many cell types are distinguished in these single‐cell studies, they repeatedly have trouble resolving the clusters of stroma and theca cell types despite using staining validation and re‐clustering of the data. The stroma compartment contributes many cells with no definitive markers, while theca cells have definitive markers but are rare. Furthermore, using single‐cell isolation techniques such as LCM that involve fixing the tissue and cutting sections of interest with a laser have the potential to disrupt the native cells and could affect the data. Despite these limitations, these studies provide us with a plethora of genes and pathways that can be explored in future analyses.

## 3 CONCLUSION AND FUTURE DIRECTIONS

The emergence of robust sequencing platforms and advanced single‐cell isolation techniques has provided the foundation for exploring the transcriptome of single cells in the human ovary. So far, applications of scRNA‐seq have focused on improving IVF outcomes and creating a comprehensive map of ovarian cell types. Now, there are a plethora of enriched gene datasets and pathway analyses to investigate as potential therapeutic or experimental targets. Due to the limited availability and precious nature of human tissue, there remains a need for repeated experiments with larger experimental groups. These reviewed single‐cell sequencing studies are limited further by their inability to deduce transcriptomic shifts through spatial relationships in the tissue. Additionally, due to the heterogeneity of the human ovary, there is an increased risk of missing unique or under‐studied cell types in sequencing experiments which is influenced by the amount of tissue received and the area within the ovary the tissue was taken from. A promising solution to these limitations involves using spatial transcriptomics methods, such as Visium or nanoString, which provide the transcriptome data of groups of cells in specific locations in the native tissue (Marx, 2021). This method has the potential to provide a transcriptome‐level understanding of features that are incompletely characterized in the ovary such as the corpora lutea, lymphatic vessels, gradients of stroma, and intermediate follicle stages. Recently, as part of the Human Cell Atlas (HCA) global initiative to create a map of every human cell type, researchers have utilized the combination of traditional scRNA‐seq with spatial transcriptomics to map several organs including: the liver, thymus, and spleen immune systems (C. Suo et al., 2022), the uterus (Garcia‐Alonso et al., 2021), and intestinal tract (Elmentaite et al., 2021). These atlases have been constructed despite similar limitations on human tissue availability and heterogeneity. For this reason, we foresee that the use of a spatial transcriptomics platform performed jointly with scRNA‐seq will help unlock a new biological understanding of the human ovary.

## AUTHOR CONTRIBUTIONS

**Jordan H. Machlin**: Writing–review and editing; writing–original draft; Investigation. **Ariella Shikanov**: Writing–review and editing.

## CONFLICT OF INTEREST

The authors declare no conflict of interest.
